# Preparing for a School-Located COVID-19 Vaccination Clinic

**DOI:** 10.1177/1942602X21991643

**Published:** 2021-02-22

**Authors:** Katherine Park, Rebecca Cartmill, Belinda Johnson-Gordon, Mary Landes, Karen Malik, Jane Sinnott, Kathy Wallace, Robin Wallin

**Affiliations:** School Nurse, Parkway School District, Chesterfield, MO; School Nurse, Parkway School District, Chesterfield, MO; School Nurse, Parkway School District, Chesterfield, MO; School Nurse, Parkway School District, Chesterfield, MO; School Nurse, Parkway School District, Chesterfield, MO; School Nurse, Parkway School District, Chesterfield, MO; School Nurse, Parkway School District, Chesterfield, MO; Director of Health Services, Parkway School District, Chesterfield, MO

**Keywords:** school-located vaccination events, school nurses, COVID-19, policy development and implementation, leadership, health promotion, community/public health

## Abstract

School-located vaccination events (SLVE) have a long history in the United States and have successfully contributed to lower morbidity and mortality due to vaccine-preventable diseases. The school is an ideal place to reach children from all cultures, socioeconomic groups, and age-groups and is conveniently situated in communities for ease of accessibility for students, parents, and staff alike. School nurses play an important role in planning for SLVE and are ideally positioned to initiate this process and provide accurate information, dispelling myths about vaccines. Because school nurses are considered a trusted source of health information by the school community, they can provide valuable education on the impact of vaccination on student and staff attendance. Conducting a successful SLVE requires research, planning, and partnerships, and these partnerships are needed both within the school setting and outside this setting, within the community at large. The proliferation of the current COVID-19 pandemic and the subsequent vaccine production has caused school nurses to take the lead in preparing for mass vaccination clinics in order to help mitigate this serious public health threat. This manuscript describes the process a group of school nurses used to develop SLVE plans in response to a pandemic.

## Introduction

In early 2020, the virus “severe acute respiratory syndrome coronavirus 2 (SARS-CoV-2),” and the disease it causes, coronavirus disease 2019 (COVID-19), was identified as a global pandemic and as of January 2021, has caused over 350,000 U.S. deaths (World Health Organization [WHO], 2021). Currently there have been two vaccines approved by the Food and Drug Administration for immediate use and as of this writing, there have been 5.3 million adults in the United States that have received initial doses of vaccines, with plans to have the majority of people fully vaccinated within the coming months (Centers for Disease Control and Prevention [CDC], 2021).

The CDC (2020b) has established clear guidance on steps to be taken when planning a vaccination clinic in a public setting, which may include school buildings. These school located clinics are commonly referred to as school-located vaccination events (SLVE), or mass-immunization clinics National Association of School Nurses (NASN, 2017). Additionally, the CDC has included recent guidance on conducting vaccination clinics during the COVID-19 pandemic, where additional considerations for social distancing, personal protective equipment use, and clinic flow are critical. Due to the complexity of conducting mass vaccination clinics, protocols have been established and recommended for such events to include planning, preclinic event activities, clinic activities, and postclinic event activities (CDC, 2020b). Once a vaccine becomes available for COVID-19, mass distribution will eventually include availability to the general public. Because this pandemic has affected our communities and caused wide-spread illness, it is important for school nurses to lead the way in creating a plan that addresses these health issues and prepares schools for the likely event that they will be provided vaccinations by the local health departments to be administered by school nurses to school staff and students.

## History and Review of the Literature

The 20th century has seen numerous pandemics. The 1918 influenza pandemic caused at least 500,000 U.S. deaths and up to 50 million deaths worldwide. Subsequent influenza pandemics in 1957 and 1968 caused at least 70,000 U.S. deaths and about 34,000 U.S. deaths, respectively. The uniqueness of the 2009 H1N1 influenza pandemic caused about 12,469 U.S. deaths but seemed to affect children more often than adults (Madhav et al., 2017).

Before the H1N1 influenza pandemic, mass vaccination events typically occurred in community settings with varying degrees of success and needed adequate staffing and strong protocols to be effective (Herman et al., 2006). Multiple reviews after the 2009 pandemic identified schools as being primary sites for administration of vaccines to children, staff, and the school community (Cho et al., 2012; Jenlink et al., 2010). While the cost of providing a mass clinic event is always a consideration and can vary widely, Cho et al. (2012) found that clinic events held at schools saved financial resources because school nurses and school staff can be utilized, eliminating the need to pay outside groups to come in and provide services. This is most effectively done during times when school is not in session, either before or after school hours or during staff development days. If providing this service during school hours for students or staff, trained school staff can help cover nurse duties to allow the nurse to focus exclusively on vaccine administration. Additionally, school nurses can be used not only to administer vaccines but also to publicize the event and procure donations for supplies, advertising, and promotion. Jenlink et al. (2010) found that by involving the right stakeholders and working closely with the local health department, a SLVE can be a very effective way to ensure mass immunization of school communities.

School districts hosting vaccine events comes with many considerations. Despite the ease of location and availability of school staff and nurses to assist with the process, there are multiple hurdles to address, all of which need to be addressed prior to conducting a mass vaccine event at school whether during or outside the school day (Lott & Johnson, 2012; see Box 1). Wilson et al. (2012) found that barriers often existed for SLVE when local school boards and the school community were not in agreement over whether schools should promote or provide vaccinations to either staff or students.

**Box 1.** Hurdles/considerations for School-Located Vaccination Events

• Ample clinic space • Adequate parking • Minimal disruption to educational schedules • Obtaining and managing signed consent forms • Language barriers • Vaccine delivery and storage • Acquisition of extra supplies • Training for staff • Adverse-reaction follow-up

Much can be learned, however, from high-performing school-based vaccination clinics to share successes and improve performance in future school-based vaccination campaigns. Klaiman et al. (2014) examined the successful implementation of 20 different vaccination events held across the United States and concluded that good communication and trusting relationships between local health department staff and the school districts were vital for the success of each successful school vaccination campaign. Additionally, flexibility was shown to be very important for schools in deciding when to hold clinics, either during school hours or after hours, as more options can increase participation. Taddio et al. (2019) specifically focused on vaccination events that were delivered to students, which helped ease the comfort levels of both students and school nurses administering vaccinations. Their small-scale study integrated the CARD System (*C*–Comfort, *A*–Ask, *R*–Relax, *D*–Distract), an intervention designed to improve the vaccination experience at schools by preparing students ahead of time for the event and allowing them to choose options they think will help them during receipt of the vaccine. Popular option choices include bringing along a stuffed toy (Comfort), asking a friend to sit with them (Ask), practicing a still yoga pose (Relax), or being allowed to use cell phone to play a video game (Distract). On completion of the first event, students were described by nurses and school staff as more prepared and less fearful during vaccinations. Nurses reported that the CARD system built on their practice; they had higher confidence in their ability to assess pain and fear and higher satisfaction with their ability to manage it. Nurses also reported improved collaboration with students and with each other.

According to the NASN (2020), school vaccination events have a long history in the United States and have successfully contributed to lower morbidity and mortality due to vaccine-preventable diseases. The school is an ideal place to reach 52 million children from all cultures, socioeconomic groups, and age-groups. Additionally, the school is conveniently located in a familiar and trusted community environment (Klaiman et al., 2014). The school also offers a convenient option for parents to have their children receive needed vaccinations without having to arrange for a healthcare provider visit or take off time from work (Fiala et al., 2013). The school nurse can play a critical role in planning a SLVE because of understanding both the needs of the community and the school.

## Identification of Need

Based on the lived experiences of many nurses currently working in our school district, we wanted to ensure we would be ready for a SLVE when a COVID-19 vaccine became available in our area. Many nurses still vividly remembered working during the 2009 influenza pandemic and wanted to improve on that process of administering vaccines at school. A group of seven nurses was assembled and began meeting weekly via Zoom starting in September. Initially, research articles on best-practice for SLVE were identified, with each nurse summarizing articles and reporting back to the others on pros, cons, and key take-a-ways. It became clear through the literature search that it would be imperative to form a partnership with our local health department, who would be the likely distributor of vaccines in our area once received from the state. A key role of school nurses is collaboration with many community partners, so the school and public health partnership is a familiar model for the delivery of healthcare in many communities. This collaboration is key to a successful clinic event (NASN, 2020).

Next, a virtual meeting was held with an official from the St. Louis County Department of Public Health (n.d.) and a list of questions, composed by the group, was presented to them about vaccine clinics in schools. Our health department currently had no agreements or partnerships with schools for providing immunizations at school, so our proposal and interest in partnering with them was important. A memorandum of agreement was written and signed that formally recognized our school district as a point of dispensing location, a place designated for mass distribution of vaccinations quickly to a large group of people.

In consultation with the Missouri Department of Health & Senior Services (n.d., 2020), the Department of Health and Human Services (USDHHS), and the St. Louis County Department of Public Health (n.d.), our group of nurses next began working on a general district vaccination plan, based on the district’s recently revised crisis and pandemic plans. In addition to background, history, and purpose, we outlined essential duties and specific actions for various school personnel, with some employees being tasked to provide backup services as needed to support the day-to-day operation and functions of the school district during clinic events. All aspects of the school day, as well as related school activities and the possibility of school being virtual at the time of vaccine availability, were addressed, and the plan requires a team effort from many departments to be successfully implemented.

One of the more important issues to be addressed during the planning phase was the concern of increased liability that could result from school vaccination programs (Pace & Dixon, 2020). Situations that could lead to increased liability include vaccinator error, adverse reactions, or unknown long-term negative side effects. Therefore, we consulted with our school district liability insurer and our district attorney prior to finalizing our plan to ensure that our district was willing to take on any potential liability. Since we were also planning to partner with our local health department, the school district would not be taking on this liability alone. Our attorney reviewed the state’s standing order and vaccine consent forms to ensure that the “hold harmless” clause in the consent forms met acceptable standards. Vaccine participants will provide written voluntary informed consent. In addition, the state standing orders included a detailed plan for vaccinators to follow to minimize risk and to track second-dose notifications. Clinical competence of vaccine administrators will be ensured by the completion of the provider training tool kits (CDC, 2020a). Local emergency medical services providers will be alerted to the vaccine clinic. Finally, all adverse reactions will be reported to the Vaccine Adverse Event Reporting System (USDHHS, 2021).

Once completed, the draft plan was forwarded to the health services director, who suggested some revisions, and then on to the superintendent’s advisory team for review and approval. It was during this time period that we became aware of the rapid acceptance of several COVID-19 vaccines by the Food and Drug Administration and the imminent initial shipments of vaccines to each state. Estimates for vaccine arrival to our health department for administration in schools was targeted for February 2021. In response, we began development of additional working documents that would help school nurses plan for and conduct a SLVE that was designed to vaccinate school staff first, since initial vaccines were indicated for the adult population only. Per our draft district plan, we had already discussed several planning assumptions. Such assumptions are based on previous research, what is known or believed to happen during SLV clinics (Lott & Johnson, 2012; Wilson et al., 2012), and can help to guide planning for other future school vaccination clinics. According to the USDHHS (2017), given the difficulty associated with estimating timing or impact, specific planning for COVID-19 vaccination clinics is based on additional assumptions, including

- delays in the availability of vaccines are likely, particularly early in the vaccine distribution period,
- the timeline and availability of a vaccine cannot be predicted with certainty,
- there may be vaccine storage and distribution barriers,
- the novel virus will have the ability to continue to spread rapidly worldwide,
- the number of those vaccinated will depend on the severity of the disease transmission at the time, and the success of public health education efforts on the importance of receiving the vaccine, and
- whether students and school staff volunteer to receive the vaccine when available cannot be predicted with certainty and may not be mandated.

**Figure 1.**
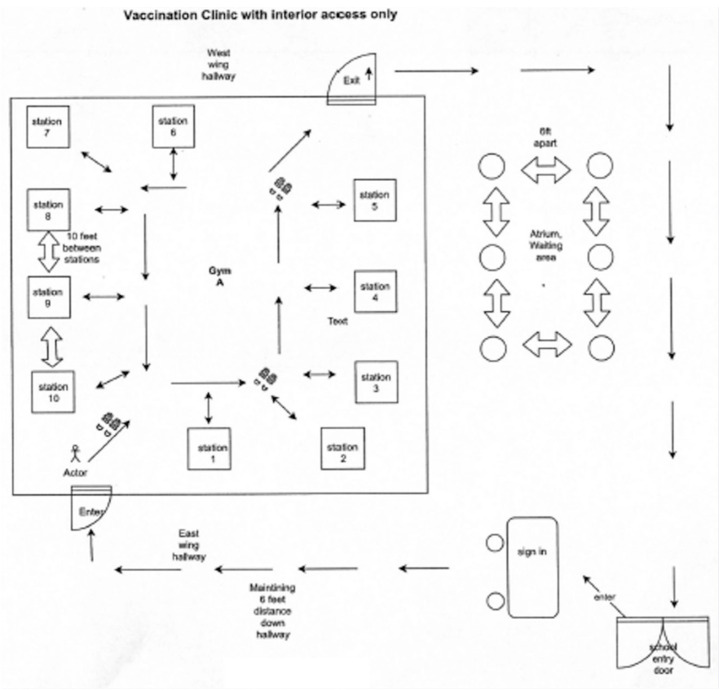
Sample School-located Vaccination Events (SLVE) Map and Layout

Based on these assumptions, our planning phase included estimating. Our initial target group of school staff and the percentage of staff likely to volunteer for vaccination, identifying centrally located buildings that will host the SLVE, creating a map and layout that can be used as a template for events (see Figure 1), and creating a checklist that can be used by the school nurses to ensure consistency during the vaccination process by outlining general operations, vaccine storage and handling, the vaccination process, and adherence to safety measures (see Table 1). A protocol was developed to ensure a skill review and assessment for the school nurses who will be administering vaccines.

**Table 1.** Checklist for SLVE Vaccine Storage and Handling.

During the clinic (Please complete each item while the clinic is occurring and review at the end of your shift).			
Vaccine storage and handling (at the facility/clinic)			
Yes	No	N.A.	
*	*		Vaccines are being kept in proper storage equipment that maintains the manufacturer-recommended temperature range.
*	*		Vaccine temperature is being monitored during the clinic. Follow the temperature monitoring guidance specified in CDC’s vaccine storage and handling toolkit: https://www.cdc.gov/vaccines/hcp/admin/storage/toolkit/storage-handling-toolkit.pdf
*	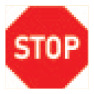 *	*	If vaccines are being stored in a storage unit at the site, vaccine temperature data are being reviewed and *documented a minimum of 2 times* during each clinic workday per vaccine manufacturer guidelines.
*	*	*	If vaccines cannot be stored in a storage unit at the site, they are being kept in a health department/manufacturer approved device.

*Note*. SLVE = school-located vaccination events; CDC = Centers for Disease Control and Prevention.

Once all clinic events have been completed, a report and program evaluation will be compiled and presented to the district to capture lessons learned from the clinic events. The evaluation measures would include looking at the process to identify and report on goal accomplishment. For example, a goal will be set to have a percentage number of people vaccinated guided by using past years’ seasonal flu vaccinations for staff. Additionally, we will work closely with our local health department and utilize their “after action” report process to evaluate our program. The After-Action Report/Improvement Plan aligns event objectives with preparedness doctrine to include the National Preparedness Goal and related framework and guidance. Event information required for preparedness reporting and trend analysis is included, and users are encouraged to add additional sections as needed to support their own organizational needs. A successful event will also produce minimal adverse reactions. Finally, recommendations will be made for future improvements and a key stakeholder meeting will convene within 1 week following the completion of the program.

## Implications for School Nursing Practice

This project has several implications for school nursing evidence-based practice. NASN’s *Framework for 21st Century School Nursing PracticeTM* (NASN, 2016) provides a helpful structure for organizing nursing interventions. The development of a SLVE plan falls under the core principle of Leadership. As change agents, we chose to improve our practice by creating a plan for vaccination clinics where none previously existed. This required us to develop new protocols and enact a system-level response, which demonstrated our value to district leadership and our school community.

The development of this vaccination plan also falls under the core principle of Community/Public Health. By educating the school community on the importance of vaccination against this novel virus, and by improving access to care in providing an on-site school-located vaccine clinic, we are performing an outreach service that addresses population-based care. Additionally, health promotion will be an extremely important component for the school nurses to focus on regardless of where people eventually choose to receive their vaccination against COVID-19.

Finally, this project also addresses the core principle of Quality Improvement. Through our research, we will be able to offer a service that will not only improve health outcomes but will also provide us with meaningful data that we can evaluate and modify for future clinic events.

Future work and research will no doubt be needed as we move forward given the unique requirements of conducting a mass vaccine clinic event that is efficient yet adherent to social distancing needs of a respiratory virus, all within the backdrop of a school setting. Expanding provision of vaccines to family and community members may be considered if resources allow. School nurses are ideally positioned to lend a voice and be key stakeholders in a plan to provide vaccinations that would likely require their skills and services. By taking initial ownership of this process, nurses can strive to preserve the continuity of essential school functions, minimize educational and social disruption, and decrease the continued spread of COVID-19.■
